# Role of calcitonin gene-related peptide in cerebral vasospasm, and as a therapeutic approach to subarachnoid hemorrhage

**DOI:** 10.3389/fendo.2012.00135

**Published:** 2012-11-15

**Authors:** Stelios Kokkoris, Peter Andrews, David J. Webb

**Affiliations:** ^1^Intensive Care Unit, Western General HospitalEdinburgh, UK; ^2^Centre for Clinical Brain Sciences, University of EdinburghEdinburgh, UK; ^3^Clinical Pharmacology Unit, British Heart Foundation Centre for Cardiovascular Science, Queen’s Medical Research Institute, University of EdinburghEdinburgh, UK

**Keywords:** GPCR, CGRP, subarachnoid hemorrhage, cerebral vasospasm, G proteins

## Abstract

Calcitonin gene-related peptide (CGRP) is one of the most potent microvascular vasodilators identified to date. Vascular relaxation and vasodilation is mediated via activation of the CGRP receptor. This atypical receptor is made up of a G protein-coupled receptor called calcitonin receptor-like receptor (CLR), a single transmembrane protein called receptor activity-modifying protein (RAMP), and an additional protein that is required for Ga_s_ coupling, known as receptor component protein (RCP). Several mechanisms involved in CGRP-mediated relaxation have been identified. These include nitric oxide (NO)-dependent endothelium-dependent mechanisms or cAMP-mediated endothelium-independent pathways; the latter being more common. Subarachnoid hemorrhage (SAH) is associated with cerebral vasoconstriction that occurs several days after the hemorrhage and is often fatal. The vasospasm occurs in 30–40% of patients and is the major cause of death from this condition. The vasoconstriction is associated with a decrease in CGRP levels in nerves and an increase in CGRP levels in draining blood, suggesting that CGRP is released from nerves to oppose the vasoconstriction. This evidence has led to the concept that exogenous CGRP may be beneficial in a condition that has proven hard to treat. The present article reviews: (a) the pathophysiology of delayed ischemic neurologic deficit after SAH (b) the basics of the CGRP receptor structure, signal transduction, and vasodilatation mechanisms and (c) the studies that have been conducted so far using CGRP in both animals and humans with SAH.

## INTRODUCTION

In the US, over 30,000 persons each year experience a subarachnoid hemorrhage (SAH). Whereas intracranial aneurysms are found in 2–5% of all autopsies, the incidence of rupture is only 2–20/100,000 individuals/year (Ingall et al., 2000). SAH is more frequent in women than men (3:2 ratio) over the age of 40, but the reverse is the case in those younger than 40 (The ACROSS Group, 2000;Ohkuma et al., 2002). Peak rupture rates occur between the ages of 50 and 60 years (The ACROSS Group, 2000;Ohkuma et al., 2002). Intracranial aneurysms account for approximately 85% of cases of non-traumatic SAH, whereas 10% have the pattern of non-aneurysmal perimesencephalic hemorrhage, a relatively harmless condition (van Gijn et al., 2007). The other causes include bleeding from other vascular malformations, moyamoya syndrome, coagulopathy, and, rarely, extension of an intracerebral hematoma (van Gijn et al., 2007). In up to 15%, no source of bleeding is identified (Kim et al., 2012). Approximately 10–15% of patients die before receiving medical treatment from the initial bleed or its immediate complications (Huang and van Gelder, 2002) and over 40% of hospitalized patients die within 1 month of the event (Ingall et al., 2000). Those that survive the initial bleed are at risk for a number of secondary insults including rebleeding (Winn et al., 1977;Ohkuma et al., 2001), hydrocephalus, and cerebral vasospasm (van Gijn et al., 2007).

Calcitonin gene-related peptide (CGRP) is one of the most potent microvascular vasodilator peptides identified to date. In the cerebral circulation, CGRP is released from sensory fibers originating in the trigeminal ganglia and acts to dilate cerebral vessels (McCulloch et al., 1986). CGRP has been found to be at least 1,000 times more potent than acetylcholine, substance P, ATP, adenosine, and 5-hydroxytriptamine, and 10–100 times more potent than the β-adrenergic agonist isoprenaline. Consequently, a dose of 15 pmol injected into human skin produces an erythema that lasts for 5–6 h (Brain et al., 1985). As we discuss later, CGRP has a particularly potent vasodilator activity in the cerebral circulation, rendering it a promising agent for the treatment of SAH-triggered cerebral vasospasm.

In the present review, we summarize the etiology and therapy of cerebral vasospasm, the biology of CGRP and its receptors, and review the role of CGRP as a treatment in SAH-associated vasospasm in both animals and humans.

## CEREBRAL VASOSPASM AFTER SAH

### DEFINITIONS

Throughout the literature, authors have used various means of defining vasospasm including terms like angiographic vasospasm, symptomatic vasospasm, and delayed cerebral ischemia (DCI). Angiographic vasospasm is a narrowing of the lumen of the major cerebral arteries, which is usually focal but may be diffuse. Vasospasm has its onset usually on day 3 after SAH, is maximal at days 6–8, and usually lasts for 2–3 weeks (Wilkins, 1990). Symptomatic vasospasm is characterized by the insidious onset of confusion and decreased level of consciousness, followed by focal motor and/or speech impairments. It is mainly a diagnosis of exclusion, when clinical deterioration occurs and hydrocephalus, rebleeding, hypoxia, and metabolic abnormalities have been ruled out. DCI is defined as symptomatic vasospasm, infarction attributableto vasospasm, or both (Frontera et al., 2009). Although about 70% of patients may develop arterial narrowing, only 30% will manifest neurological deficits. The outcome of DCI itself is death in about one-third and permanent deficit in another third (Dorsch, 1995). In the present review the term vasospasm is defined as arterial vessel narrowing.

### VASOSPASM PATHOPHYSIOLOGY

#### Nitric oxide

Loss of the biological effect of nitric oxide (NO) is considered to play a pivotal permissive role in the development of cerebral vasospasm. The principal effect of NO on cerebral vessels is the relaxation of vascular smooth muscle cells, with decreased bioavailability of NO being implicated in the formation of SAH-induced vasospasm. The depletion of NO has been assumed to occur via several mechanisms in the setting of SAH. First, due to its high affinity for hemoglobin (Hb), NO is scavenged by Hb released during the breakdown of subarachnoid blood (Goretski and Hollocher, 1988;Ignarro, 1990). Second, it is possible that the production of NO is decreased in SAH, as a result of the down-regulation of endothelial NO synthase (eNOS) and neuronal NOS (nNOS;Pluta, 2005). This is supported by studies that revealed the down-regulation/dysfunction of eNOS, and loss of nNOS in spastic arteries after SAH (Hino et al., 1996;Pluta et al., 1996), as well as the finding that levels of asymmetric dimethylarginine (ADMA), an endogenous inhibitor of eNOS, are elevated in the setting of cerebral vasospasm (Jung et al., 2004). Third, NO may reverse the effects of the potent vasoconstrictor endothelin-1 (ET-1;Thomas et al., 1997). Therefore, in the setting of decreased NO levels, the balance of vasodilator and vasoconstrictor influences is altered, and the relatively increased actions of ET-1 can potentiate cerebral vasospasm.

#### Endothelin-1

ET-1 is an extremely potent vasoconstrictor. In the brain, it is primarily produced by endothelial cells in response to ischemia, though it can also be produced by neurons, astrocytes, and activated leukocytes (Fassbender et al., 2000;Chow et al., 2002;Dumont et al., 2003). Levels of ET-1 are high in the plasma and cerebrospinal fluid (CSF) of SAH patients, correlate with the persistence of cerebral vasospasm (Seifert et al., 1995;Juvela, 2000), and decline in the absence of vasospasm (Seifert et al., 1995). Conversely, the administration of ET-1 antagonists or endothelin converting enzyme inhibitors prevents vasospasm (Kwan et al., 2002;Macdonald et al., 2008). Lastly, ET-1 induces NADPH oxidase expression and oxidative stress in human endothelial cells (Duerrschmidt et al., 2000).

#### Inflammation

Expression of adhesion molecules facilitates leukocyte adherence to the endothelium. Adhesion molecules, such as ICAM-1, VCAM-1, and E-selectin, have been found to be elevated in the CSF of patients with SAH and in blood vessel walls exposed to clot (Polin et al., 1998;Dumont et al., 2003). Leukocytes can contribute to vasospasm by promoting free radical formation that may evoke endothelial dysfunction (Grisham et al., 1998;Sullivan et al., 2000), and by producing a variety of vasoactive substances, including ET-1 and cytokines (Fassbender et al., 2000). Several cytokines have been found to be up-regulated in cerebral vasospasm, including TNF-alpha, IL-1, IL-6, and IL-8 (Hirashima et al., 1997;Fassbender et al., 2001;Takizawa et al., 2001).

#### Oxidative stress

Oxyhemoglobin (OxyHb) may catalyze generation of reactive oxygen species (ROS). Free radicals are considered to play a pivotal role in cerebral vasospasm through various mechanisms. First, they can initiate lipid peroxidation, whose products, lipid peroxides, are capable of producing vasospasm and damaging the structure of arteries (Lin et al., 2006). Second, it has been hypothesized that ROS can activate the protein kinase C (PKC) pathway directly and indirectly, through enhancement of the metabolism of membrane phospholipids resulting from peroxidative damage. This, in turn, can lead to vasospasm (Asano and Matsui, 1999). Other possible vasoactive compounds are bilirubin oxidation products (BOXes). Once bilirubin is formed, it is subsequently oxidized into BOXes, reaching maximum concentrations during the peak vasospasm period of 4–11 days. They are thought to be potentiators of cerebral vasospasm once it has been initiated, rather than primary initiators (Clark and Sharp, 2006).

#### Hemoglobin

A large body of evidence suggests that OxyHb, the ferrous form of hemoglobin, released from lysed erythrocytes, is a mediator of vasospasm. More specifically, OxyHb causes prolonged contraction of isolated cerebral arteries (Toda et al., 1991), and intracisternal injections of this agent result in cerebral vasospasm (Macdonald et al., 1991). Indeed, the presence of OxyHb in the CSF of patients after SAH and the extent of hemorrhage are correlated with the distribution, severity, and time course of vasospasm (Mayberg et al., 1990). Ferrous hemoglobin released from subarachnoid clot could lead to delayed arterial narrowing by a number of mechanisms, such as scavenging or decreased production of NO (Pluta, 2005), free radical production, modification of K^+^ and Ca^2+^ channels (Ishiguro et al., 2008), differential up-regulation of genes (Vikman et al., 2006), and activation of the Rho/Rho kinase and PKC pathways (Wickman et al., 2003).

#### Intracellular Ca^2+^

Vasospasm can be regarded as an abnormal and prolonged contraction of vascular smooth muscle. The intracellular free Ca^2+^ level plays a pivotal role in the regulation of smooth muscle contractility (Horowitz et al., 1996). Following SAH, changes have been reported in the electrical properties of smooth muscle cells of small diameter cerebral arteries leading to enhanced Ca^2+^ influx, vasoconstriction, and decreased cerebral blood flow (Koide et al., 2011). Cerebral arteries from healthy animals express only L-type voltage-dependent Ca^2+^ channels. Expression of an additional type of voltage-dependent Ca^2+^ channels (R-type) occurs after SAH, leading to increased Ca^2+^ channel density, increased Ca^2+^ influx, and vasoconstriction (Ishiguro et al., 2005).

#### Cortical spreading depolarization

This is a pathogenetic process that has attracted much attention lately. The term “cortical spreading depolarization” describes the wave of near-complete neuronal depolarization and neuronal swelling in the brain that is ignited when passive cation influx across the cellular membranes exceeds ATP-dependent Na^+^ and Ca^2+^ pump activity. The cation influx is followed by water influx and shrinkage of the extracellular space by ~70% (Dreier et al., 2009). Although the ignition of cortical spreading depolarization occurs passively, driven by electrical and diffusion forces, energy consumption paradoxically increases since Na^+^ and Ca^2+^ pumps are immediately activated to correct the intracellular Na^+^ and Ca^2+^ surge. As a consequence, regional cerebral blood flow increases during the neuronal depolarization phase. The opposite of this physiological hemodynamic response to cortical spreading depolarization is termed “the inverse hemodynamic response,” and occurs when there is local dysfunction of the microvasculature. With the inverse response, severe microvascular spasm instead of vasodilatation is coupled to the neuronal depolarization phase, and the term “cortical spreading ischemia” describes the cortical spreading depolarization-induced perfusion deficit (Dreier et al., 2009).

#### Neurogenic factors

The cerebral arteries have sympathetic, parasympathetic, and sensory innervation. It has been postulated that SAH causes a derangement of neuronal regulatory mechanisms, which in turn leads to vascular smooth muscle contraction. The vasoconstriction is associated with a decrease in CGRP levels in cerebral perivascular nerves (Edvinsson et al., 1991) and an increase in CGRP levels in blood draining from the external jugular vein (Juul et al., 1990), suggesting that CGRP is released antidromically from trigeminal sensory perivascular nerves to oppose the vasoconstriction. This evidence has led to the concept that administration of CGRP may be beneficial in SAH-associated vasospasm. The molecular characteristics of CGRP and its use as a treatment option in SAH are reviewed in Sections “Calcitonin Gene-related Peptide Biology” and “Calcitonin Gene-related Peptide and SAH,” respectively, of the present article.

### TREATMENT OF VASOSPASM

The management of vasospasm involves routine “prophylactic” measures as well as more aggressive interventions, reserved for situations where there are signs or symptoms of DCI.

#### Hemodynamic therapy

The use of triple-H therapy (hypervolemia, hypertension, and hemodilution) stems from numerous clinical observations noting improvement in patients’ clinical symptoms following induced hypertension and volume expansion (Kosnik and Hunt, 1976;Kassell et al., 1982). The relative contribution of each component is debated. However, there are many uncertainties for the use of prophylactic hemodynamic therapy following SAH. Two studies randomly assigned normovolemic or hypervolemic therapy to patients and reported no difference in the incidence of DCI between groups (Lennihan et al., 2000;Egge et al., 2001).

#### Nimodipine

Nimodipine is safe, cost-effective, and reduces the risk of poor outcome and secondary ischemia (Neil-Dwyer et al., 1987;Welty, 1987;Kostron et al., 1988;Mee et al., 1988), but has very modest effects. It is used prophylactically in all patients with SAH. Its precise mechanism of action remains unclear. Despite being shown to reduce the incidence of DCI and cerebral infarction in clinical trials, it has negligible effects on angiographic vasospasm; nimodipine may be neuroprotective by blocking Ca^2+^ influx at a neuronal level (Al-Tamimi et al., 2010).

#### Intracisternal thrombolysis

A meta-analysis looking at a total of 652 patients who were treated with intracisternal thrombolytics concluded that thrombolytic therapy had a statistically significant beneficial effect. However, the authors acknowledged the lack of large, randomized prospective trials (Amin-Hanjani et al., 2004).

#### Endovascular techniques

Endovascular techniques frequently play a role in the aggressive treatment of vasospasm. They include transluminal angioplasty and intra-arterial infusion of vasodilators (papaverin, nicardipine, verapamil, etc.;Brisman et al., 2006). Transluminal balloon angioplasty is very effective at reversing angiographic spasm of large proximal vessels and produces a sustained reversal of arterial narrowing (Brisman et al., 2006;Jestaedt et al., 2008). The optimal timing of angioplasty in relation to medical therapy is uncertain. Major complications occur in ~5% of procedures and include vessel rupture, occlusion, dissection, hemorrhagic infarction, and hemorrhage from unsecured aneurysms (Zwienenberg-Lee et al., 2006).

#### Statins

Statins have been shown to possess cholesterol-lowering-independent pleiotropic effects in different clinical settings, including a decrease in the incidence and duration of severe vasospasm as well as a reduction in the mortality rate after SAH (Lynch et al., 2005;Tseng et al., 2005, 2007). Statins are thought to be beneficial in the prevention of cerebral vasospasm by down-regulating inflammation and up-regulating the expression of eNOS and therefore NO (Sugawara et al., 2011).

#### Other treatments

Clazosentan, an endothelin receptor A (ET_A_) antagonist decreased the incidence of severe vasospasm, DCI and new infarcts seen on CT scans in a dose-dependent fashion. However, CONSCIOUS 1 study (a phase 2 trial) did not show a reduction in patient mortality, though the study was underpowered for this endpoint (the primary end point of this study was moderate or severe vasospasm within 14 days;Macdonald et al., 2008). CONSCIOUS 2 study (a phase 3 trial) included 1157 patients and its primary composite end point comprised all-cause mortality and vasospasm related morbidity. This study showed that clazosentan at 5 mg/h had no significant effect on mortality and vasospasm-related morbidity or functional outcome (Macdonald et al., 2011).

Erythropoietin (EPO) has also been examined in the setting of cerebral vasospasm. Apart from being potentially neuroprotective, EPO may play a role in preventing vasospasm by increasing the phosphorylation of eNOS (Santhanam et al., 2005), a potentially important mechanism for increasing NO production.

A recent randomized controlled trial (MASH 2) including 1204 patients did not show any benefit from intravenous (i.v.) magnesium sulfate administration in clinical outcome after aneurysmal SAH (Dorhout Mees et al., 2012).

Other drugs under investigation are tirilazad, a free radical scavenger (Haley et al., 1997), fasudil, a Rho-kinase inhibitor that inhibits vascular smooth muscle contraction (Shibuya et al., 1992), sodium nitrite, an NO donor (Pluta et al., 2005) and cisternal placement of prolonged-release nicardipine-loaded polymers (Kasuya et al., 2005).

## CALCITONIN GENE-RELATED PEPTIDE BIOLOGY

### CALCITONIN GENE-RELATED PEPTIDE

Calcitonin gene-related peptide is expressed in a subgroup of small neurons in the dorsal root, trigeminal, and vagal ganglia, which respond to noxious, thermal, or visceral input. These peptidergic neurons use L-glutamate as their primary neurotransmitter and project to the dorsal horn, trigeminal nucleus caudalis, or nucleus of the solitary tract. CGRP increases neurotransmitter release and neuronal responsiveness to noxious stimulation at all these levels, which leads to central sensitization underlying chronic pain states (Benarroch, 2011). CGRP can also be released antidromically in the periphery, eliciting vasodilatation as a component of neurogenic inflammation. CGRP may be involved in the pathophysiology of inflammatory and neuropathic pain. Involvement of CGRP in migraine headache has led to the development of CGRP antagonists for treatment of this disorder (Benarroch, 2011).

**FIGURE 1:**
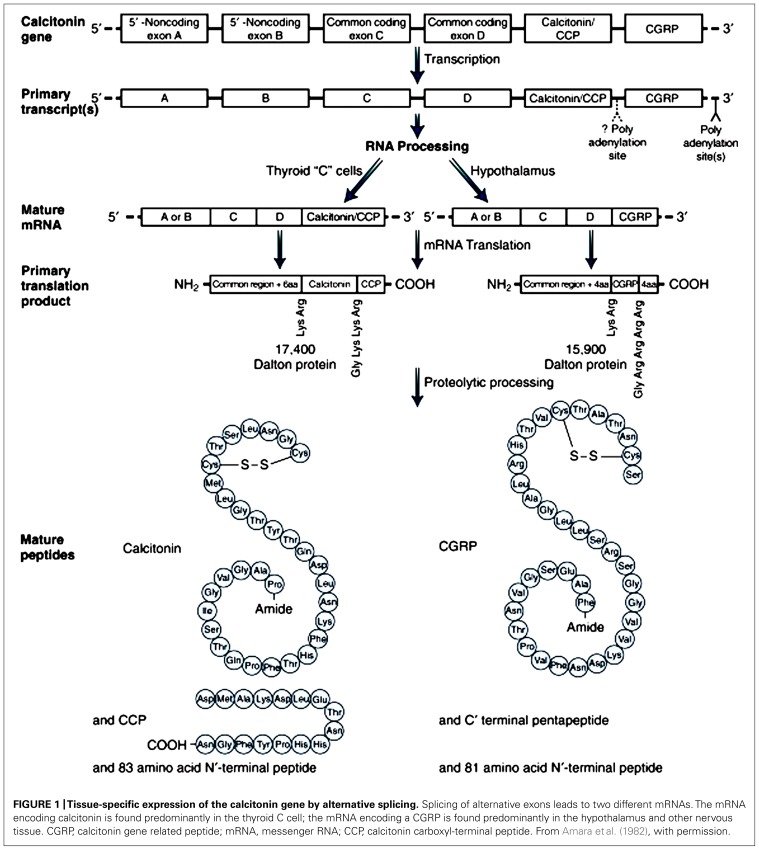
**Tissue-specific expression of the calcitonin gene by alternative splicing**.Splicing of alternative exons leads to two different mRNAs. The mRNA encoding calcitonin is found predominantly in the thyroid C cell; the mRNA encoding a CGRP is found predominantly in the hypothalamus and other nervous tissue. CGRP, calcitonin gene related peptide; mRNA, messenger RNA; CCP, calcitonin carboxyl-terminal peptide. FromAmara et al. (1982), with permission.

Calcitonin gene-related peptide is a 37-amino acid neuropeptide that was identified in 1982 by molecular biological techniques in the thyroid of aging rats and medullary thyroid carcinomas in humans, which were found to contain an alternative peptide product from the calcitonin gene (Amara et al., 1982). CGRP, in common with other members of this peptide family, is derived from the calcitonin gene. Other members of this family include adrenomedulin (AM), which is a potent vasodilator, amylin (AMY), which is important for maintaining glycemic control, and calcitonin, which contributes to calcium metabolism (Hay, 2007). CGRP exists in two forms, named αCGRP and βCGRP. While these two isoforms share the same biological activities, and differ by only three amino acids in the human (Steenbergh et al., 1985, 1986), they are formed from two distinct genes, which share >90% homology, at different sites on chromosome 11. CALC I gene forms calcitonin and αCGRP, whereas CALC II forms βCGRP (Alevizaki et al., 1986). αCGRP synthesis is caused by alternative splicing of the calcitonin gene (Amara et al., 1982; **Figure 1**). βCGRP is known to be transcribed from its own distinct gene (Steenbergh et al., 1985, 1986). The majority of CGRP within the body is αCGRP and primarily expressed in the peripheral and central nervous system. βCGRP is mainly expressed in the gut (Mulderry et al., 1988). However, it has also been identified in the central nervous system, pituitary, thyroid, and in medullary thyroid carcinoma as a major CGRP form together with αCGRP (Petermann et al., 1987).

Data from NMR studies suggest that CGRP consists of a characteristic N-terminal disulfide bridge-linked loop between cysteines Cys_2_ and Cys_7_, followed by an alpha-helix in amino acids Val_8_-Arg_18_ (Breeze et al., 1991). The next domain at residues 19–27 forms a hinge region (Conner et al., 2002). The C-terminus lies at residues 28–37, and contains two turn regions which form a putative binding epitope (Carpenter et al., 2001). It appears that the N-terminal cyclic portion of the CGRP molecule, containing a ring structure with a disulfide bond, is essential for agonistic activity (Maggi et al., 1990). It is interesting to note that the C-terminal fragment, CGRP_8–37_, is devoid of any agonist activity at CGRP receptors, although it behaves as a competitive antagonist against the intact peptide (Chiba et al., 1989).

Calcitonin gene-related peptide is widely distributed in the central and peripheral nervous systems, primarily in sensory fibers that are closely associated with blood vessels (Uddman et al., 1986). CGRP is often co-localized with other peptides in these fibers, especially the tachykinin substance P (Uddman et al., 1986). In the cerebral circulation, CGRP is released from sensory fibers originating in the trigeminal ganglia and acts to dilate cerebral vessels (McCulloch et al., 1986). In the gut, CGRP is also released from spinal afferents, where it dilates mucosal blood vessels and may protect against the acidic environment (Holzer, 2000). CGRP-containing fibers also innervate coronary arteries of the heart (Gulbenkian et al., 1993).

The regulation of CGRP production is poorly understood. At a cellular level, nerve growth factor (NGF) up-regulates CGRP via the Ras/Raf/mitogen-activated protein kinase kinase-1 (MEK-1)/p42/p44 pathway (Freeland et al., 2000).

In the human circulation, CGRP has a half-life of approximately 7–10 min (Kraenzlin et al., 1985;Struthers et al., 1986). Regarding its metabolism, it seems that there is not an obvious mechanism, and it is probably broken down via a number of routes. First, mast cell tryptase has a potent effect in cleaving CGRP into inactive fragments, both *in vivo* and *in vitro*. More specifically, if both CGRP and substance P are released simultaneously, then CGRP could be inactivated by enzymes (tryptases), released by mast cells in response to substance P. This mechanism has been demonstrated in skin (Brain and Williams, 1988, 1989). Second, a matrix metalloproteinase II has the ability to metabolize CGRP and remove its vasodilator activity (Fernandez-Patron et al., 2000). Third,Sams-Nielsen et al. (2001) have provided evidence that CGRP is taken back up into sensory nerve terminals after repolarization *in vitro*. Finally, in the CSF, αCGRP is degraded by an endopeptidase that cleaves the peptide at the Leu_*1*6_-Ser_17_ bond (Le Greves et al., 1989).

### STRUCTURE OF CGRP RECEPTORS

Many peptides, including the CGRP family, mediate their actions via G protein-coupled receptors (GPCR). The GPCRs form the largest family of cell-surface proteins that are capable of interacting with an extracellular stimulus and transducing that stimulus to produce a reaction inside a cell (Pierce et al., 2002). All GPCRs have seven transmembrane spanning domains, an extracellular N-terminus and an intracellular C-terminus and can be divided into three families based on signature amino acid sequences. Family A is the largest and generally binds small molecules and short peptides. Receptors in this class have been studied extensively, including photoreceptor rhodopsin, as well as adrenergic and olfactory receptors. Family B receptors bind larger peptides in the range of 27 to approximately 50 amino acids (secretin, glucagons, VIP, etc.). These receptors mediate the actions of CGRP and related peptides (Poyner et al., 2002;Hoare, 2005). Family C receptors include glutamate and GABA_B_ receptors (Pierce et al., 2002).

**FIGURE 2:**
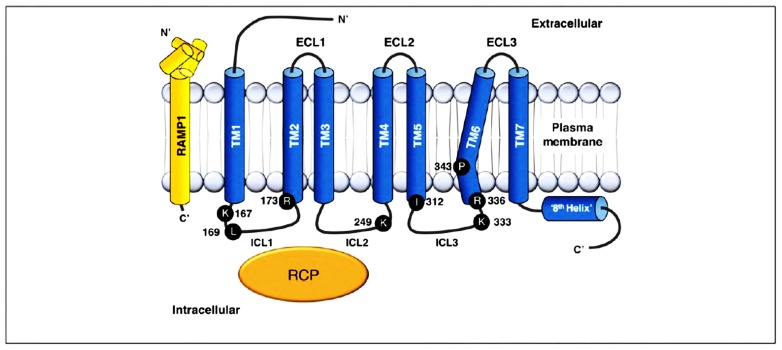
** Structure of CGRP receptor**. CGRP receptor components and important residues for receptor signaling and internalization. The CGRP receptor is formed by CLR (blue), RAMP1 (yellow), and RCP (orange). Functionally important residues are shown as single letter abbreviations. CGRP, calcitonin gene-related peptide; CLR, calcitonin receptor-like receptor; RAMP, receptor activity-modifying protein; RCP, receptor component protein; C′, C-terminal; EC, extracellular loop; ICL, intracellular loop; N′, N-terminal; TM, transmembrane. FromWalker et al. (2010), with permission.

Calcitonin receptor-like receptor (CLR), which belongs to family B of the GPCRs, comprises the main functional unit of the CGRP receptor (**Figure 2**). It was not until McLatchie’s work (McLatchie et al., 1998) was published that it was recognized that a novel family of single transmembrane domain proteins, called receptor activity-membrane proteins (RAMP), were required to allow CLR to bind peptide and transduce signal. Three RAMPs have been identified so far (RAMP_1_, RAMP_2_, and RAMP_3_). Each RAMP has a single transmembrane-spanning domain, a short intracellular C-terminal tail (~9 amino acids) and a long extracellular-terminus (~100 amino acids;McLatchie et al., 1998). As a result of CLR and calcitonin receptor (CTR) interactions with RAMP, the International Union of Pharmacology (IUPHAR) nomenclature recognizes that CGRP interacts with CLR/RAMP_1_ (CGRP_1_) receptors, whereas AM interacts with CLR/RAMP_2_ (AM_1_) or CLR/RAMP_3_ (AM_2_) receptors. The CTR without RAMP is sufficient for calcitonin binding, but CTR with RAMP 1, 2, or 3 are AMY_1_, AMY_2_, and AMY_3_ receptors, respectively (Poyner et al., 2002). The discovery of RAMPs has led to evolution of our understanding of how receptor diversity is implemented, providing a novel mechanism for generating receptor subtypes within a subset of family B GPCRs (Sexton et al., 2006).

The primary function of CLR is thought to be related to ligand binding, whereas the RAMP molecule plays a crucial role in receptor trafficking to the membrane and determination of receptor pharmacology. The RAMP family regulate the glycosylation and transport of the CLR. However, they are not CGRP receptors by themselves (McLatchie et al., 1998;Sexton et al., 2009). Terminal glycosylation of the receptor and transit from the endoplasmic reticulum/Golgi apparatus to the cell surface require interaction of CLR with RAMP (Sexton et al., 2009).

Calcitonin gene-related peptide receptor activation is known to involve several crucial elements, in common with other GPCRs, such as the presence of a proline “kink” in transmembrane helix (TM)6 (Conner et al., 2005), and a putative ‘DRY’ motif equivalent (Conner et al., 2007), similar to family A GPCRs. There is also evidence suggesting stabilization of the CLR interaction with G “alpha” s (Ga_s_) by another 17kDa intracellular membrane protein, called RCP (Evans et al., 2000).

The existence of two receptors, CGRP_1_ and CGRP_2_, was originally proposed in the late 1980s, with the CGRP_1_ receptor being the predominant mediator of cardiovascular effects. This receptor classification was developed as a consequence of pharmacological studies carried out with different agonists and antagonists in a range of tissue preparations, especially the positive inotropic effect in the guinea pig or rat atrium for determination of CGRP_1_ receptor activity, and the inhibition of electrically evoked twitch responses in the rat vas deferens for determination of CGRP_2_ receptor activity (Dennis et al., 1989, 1990;Dumont et al., 1997). In general, receptors that can be antagonized by the 30-amino acid fragment of CGRP, CGRP_8–37_, with an approximate pA_2_ value of 7.0 are designated as CGRP_1_ receptors, while those that CGRP_8–37_ block with a pA_2_ of 6.0 or less are classified as CGRP_2_ receptors (Quirion et al., 1992;Poyner, 1995). However, it is questionable whether the CGRP_2_ receptor is a single receptor type or whether it is, in fact, explained by multiple molecular entities (Hay, 2007). In contrast, CGRP_1_ is a well-defined receptor type consisting of CLR and RAMP_1_.

### SIGNAL TRANSDUCTION OF CGRP RECEPTOR

**FIGURE 3:**
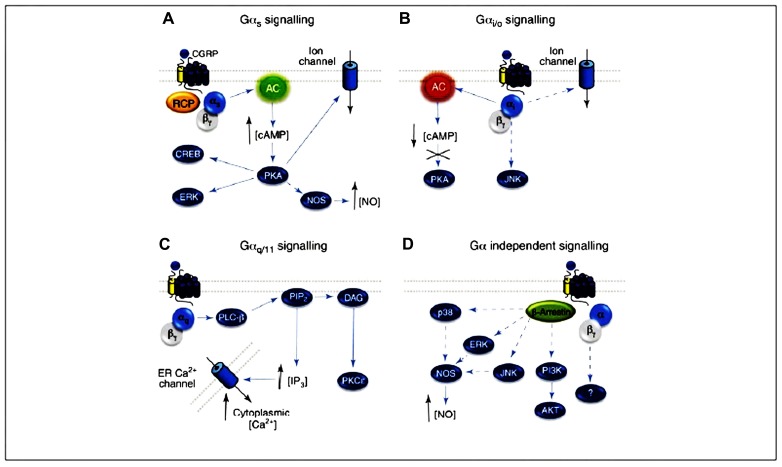
**CGRP receptor-mediated intracellular signaling**. **(A)** Ga_s_ signaling increases AC (green) activity, elevating intracellular cAMP, activating PKA and subsequently many potential downstream effectors. **(B)** The CGRP receptor might also couple to Ga_i/o_, reducing AC (red) activity, decreasing intracellular cAMP and reducing PKA activity. **(C)** CGRP signaling via Ga_q_ activates PLC-b, which cleaves PIP_2_ into IP_3_ and DAG, resulting in elevated intracellular Ca^2+^ and PKC activation. **(D)** The CGRP receptor might also utilize Ga-independent signaling, and G_β__γ_- or b-arrestin-mediated signaling pathways. Arrows represent reported pathways; broken arrows represent potential or inferred pathways. CGRP, calcitonin gene-related peptide; CLR, calcitonin receptor-like receptor; Gα, α subunit of the G protein; NO, nitric oxide; NOS, nitric oxide synthase; AC, adenylyl cyclase; cAMP, cyclic adenosine monophosphate; PKA, protein kinase A; PKC, protein kinase C; RCP, receptor component protein; AC, adenylate cyclase; ER, endoplasmic reticulum; PIP_2_, phosphatidylinositol 4,5-bisphosphate; DAG, diacylglycerol. FromWalker et al. (2010), with permission.

Several mechanisms involved in CGRP-mediated vasorelaxation have been identified. These mechanisms include either NO-dependent endothelium-dependent mechanisms or cAMP-mediated endothelium-independent pathways. The most common pathway is NO- and endothelium-independent. Activation of the CGRP receptor is generally accepted to result in Ga_s_-mediated activation of adenylate cyclase, with a subsequent increase in cAMP and activation of protein kinase A (PKA). In the absence of endothelium, CGRP is able to cause relaxation, suggesting it must directly act on the smooth muscle cells to stimulate adenylate cyclase (Edvinsson et al., 1985, 1998;Crossman et al., 1990). The resulting rise in cAMP then activates PKA, which phosphorylates and opens up ATP-sensitive K^+^ channels, thus leading to relaxation (**Figure 3A**;Nelson et al., 1990).

Endothelium-independent relaxation to CGRP occurs in the majority of tissues examined to date. Exceptions include the rat aorta, where the relaxation to CGRP occurs only in the presence of an intact endothelium and is attenuated by inhibitors of NO synthase, implying an NO-dependent mechanism (Brain et al., 1985;Gray and Marshall, 1992a,b). A significant increase in both cAMP and cGMP occurs and is also dependent on the presence of endothelium (Gray and Marshall, 1992a). This implicates the release of NO from the endothelium, which then relaxes the smooth muscle cells through activation of guanylate cyclase and accumulation of cGMP. Moreover, it has been shown that cAMP is able to stimulate eNOS activity, leading to increased synthesis and release of NO (Ferro et al., 1999;Queen et al., 2000). The activation of eNOS via cAMP is probably mediated via PKA, as a study demonstrated that various protein kinases can phosphorylate and activate eNOS (Butt et al., 2000). It is a possibility that CGRP causes an increase in cAMP in endothelial cells, which leads to PKA activation. PKA, in turn, activates eNOS, which results in NO release, and thus relaxation of the smooth muscle (**Figure 3A**).

There is some evidence for Ga_i/o_ signaling by the CGRP receptor, which is traditionally identified by sensitivity to pertussis toxin (PTX; **Figure 3B**). The CGRP-mediated stimulation of Ca^2+^ transients in rat nodose neurons and the activation of c-Jun N-terminal kinase (JNK) in SK-N-MC cells (which express endogenous CGRP receptors) both displayed PTX sensitivity (Wiley et al., 1992;Disa et al., 2000).

The CGRP receptor may also be able to stimulate intracellular activity through a different G protein.Aiyar et al. (1999) reported that CGRP was able to activate phospholipase C (PLC) in HEK293 cells, leading to an increase in intracellular Ca^2+^ via inositol trisphosphate (IP3) activity. This increase in Ca^2+^ occurred concurrently with the stimulation of adenylyl cyclase and accumulation of cAMP. Activation of PLC is considered to occur through G_q/11__α_, rather than through Gα_s_, suggesting that the activated CGRP receptor is able to interact with both types of G protein. If this mechanism is present in endothelial cells, it provides an alternative explanation for CGRP activation of eNOS (which is traditionally considered to be dependent on Ca^2+^/calmodulin for activation), independently of cAMP accumulation. The possibility that CGRP receptors may be coupled to phosphatidylinositol turnover is supported by another study that found this secondary messenger pathway in skeletal muscle (Laufer and Changeux, 1989; **Figure 3C**).

Recently,Meens et al. (2012) reported that activated CGRP receptors induce cyclic nucleotide-independent relaxation of vascular smooth muscle cells in mesenteric resistance arteries and terminate arterial effects of ET-1 via G_β__γ_. More specifically, CGRP receptor activation causes cAMP production but the relaxation of rat mesenteric resistance arteries induced by activation of this receptor involves G_β__γ_ and is not dependent on cAMP (**Figure 3D**).

Another study byMeens et al. (2010) discovered that CGRP released from peri-arterial sensory motor nerves terminates long-lasting vasoconstrictor effects of ET-1 by promoting dissociation of ET-1/ET_A_-receptor complexes.

The CGRP receptor can also potentially activate other downstream signaling molecules, such as PKC and mitogen-activated protein kinase (MAPK) cascades, such as p38, JNK, and extracellular receptor activated kinase 1/2 (ERK ½;Walker et al., 2010). CGRP receptor signaling is regulated by desensitization, internalization, and trafficking, which, as with other GPCRs, involves GPCR kinases (GRK), β arrestin, and clathrin- and dynamin-dependent endocytosis (Walker et al., 2010).Padilla et al. (2007) proposed a mechanism by which endosomal endothelin converting enzyme-1 (ECE-1) degrades CGRP in endosomes to disrupt the peptide/receptor/β-arrestin complex, freeing internalized receptors from β-arrestins and promoting recycling and resensitization, resulting in long-lasting vascular relaxing response to CGRP.

## CALCITONIN GENE-RELATED PEPTIDE AND SAH

### PRELIMINARY OBSERVATIONAL STUDIES

An animal study of experimental SAH in rats revealed that the sensory innervation of the cerebral circulation by CGRP-containing fibers appeared to be reduced after SAH (estimated by the number of fibers present), and there was also a larger vasodilating response to CGRP in basilar arteries after SAH as compared to vessels from control animals. The reduction in CGRP could be due to release of the transmitter from the perivascular nerve terminals caused by blood in the subarachnoid space (Edvinsson et al., 1990). 

In another study (Edvinsson et al., 1991), the proximal parts of the middle cerebral artery (MCA) were collected within 24 h after death from five humans suffering SAH (5–10 days beforehand) and from six subjects dying from myocardial infarction. In humans who had died from SAH the level of CGRP was nearly not detectable, being in contrast to that seen in age and sex matched subjects who had died of myocardial infarction. The trigemino-cerebrovascular system was suggested by the authors to act as an anti-vasoconstrictor system by releasing stored peptides, CGRP being the most likely candidate.

Juul et al. (1995) measured CGRP levels with specific radioimmunoassays (RIA) in patients with SAH, after operation with aneurysm clipping and nimodipine treatment. They used samples taken either from the external jugular vein (*n* = 20) or from the CSF (*n* = 14) during the postoperative course. They also used samples from healthy volunteers. The degree of vasoconstriction in the patients was monitored with Doppler ultrasound recordings. CGRP concentrations from the external jugular vein were significantly higher than from controls. Also, the CGRP level was measurable in SAH CSF but not in CSF of controls.

Others (Tran Dinh et al., 1994) showed that the basal level of endogenous CGRP in CSF was 0.77 nmol/L in rabbits. The CGRP concentration peaked at 14 nmol/L within 30 min, and at 8 nmol/L within 24 h, after SAH. They further showed that 3 days after SAH the CGRP concentration in CSF declined to 3.5 nmol/L.

Nozaki et al. (1989a) produced a model of SAH by a single injection of fresh autologous arterial blood into the cisterna magna of dogs. Then, they examined changes of CGRP immunoreactivity immunohistochemically in perivascular nerve fibers of the large pial arteries. CGRP in cerebrovascular nerve fibers was suppressed after SAH. The suppression was first detected on the third day after SAH, and was most marked during the 7th to 14th day. CGRP, however, recovered to a normal level by the 42nd day after SAH.

Arienta et al. (1991) isolated the basilar artery from five rabbits subjected to SAH and five control animals. A mild or severe vasospasm was observed in the basilar artery about 15 min after injection of blood in the cisterna magna, while fluorescence immunohistochemistry revealed a marked decrease of the perivascular nerves containing CGRP in the animals of the experimental group, as compared to the control group.

### EFFECTS OF CGRP ADMINISTRATION ON CEREBRAL VASOSPASM AFTER EXPERIMENTAL SAH IN ANIMALS (Table 1)

**Table 1:** Studies of CGRP administration after experimental SAH in animals.

Reference	Animal model	Total sample size (intervention/ control)	Way of CGRP administration	CGRP dose	Results	Adverse events
Nozaki etal. (1989b)	Dog	30 (22/8)	i.c.	2 × 10^–10^ mol/kg	Vasospasm was reversed completely	AP and HR slightly increased
Imaizumi etal. (1996)	Rabbit	16 (8/8)	i.c.	10^–10^ mol/kg	Basilar artery dilated from 73 to 117%, significantly larger than 67% in control (*p* < 0.01)	None
Toshima etal. (1992)	Rabbit	41 (17/24)	i.c./i.v.	100 ng/kg/min i.c./100 ng/kg/min i.v.	Basilar artery diameter in either i.v. or i.c. CGRP groups was significantly greater than that of the respective control group	AP drop in i.v. CGRP administration
Ahmad etal. (1996)	Rabbit	45 (22/23)	i.c. slow-release tablet	24 or 153 μg	Vasospasm was completely reversed	None
Inoue etal. (1996)	Monkey	10 (5/5)	i.c. slow-release tablet	1,200 μg	Cerebral vasospasm was significantly ameliorated	None
Toyoda etal. (2000)	Rabbit	16 (8/8)	i.c. adenovirus-mediated CGRP gene transfer	NA	Arterial diameter was similar before and after SAH in CGRP group	None
Satoh etal. (2002)	Dog	20 (8/12)	i.c. adenovirus-mediated CGRP gene transfer	NA	Vasospasm was significantly reduced compared with the control group	None

*i.c., intra-cisternal; i.v., intravenous; AP, arterial pressure; HR, heart rate; SAH, subarachnoid hemorrhage; NA, non-applicable.*

Nozaki et al. (1989b) produced experimental SAH in 30 dogs by injecting autologous arterial blood into the cisterna magna. They used two models of injection: in the first, single-injection model, 1 ml/kg of blood was injected on day 0, while 0.5 ml/kg of blood was injected successively 48 h apart in the second, double-injection model, on day 0 and day 2. The diameter of the basilar artery was measured by angiography. The most marked constriction of the basilar artery was seen on day 3 after SAH in the single-injection model and on day 7 in the double-injection model. When 10^–10^ mol/kg of CGRP was administered intracisternally (i.c.) on day 3 in the single-injection model, cerebral vasospasm reversed completely. The effect began to appear 5 min after CGRP administration, continued for 4 h, and disappeared by 24 h after the administration. When CGRP was administered at doses of 10^–11^ to 2 × 10^–10^ mol/kg on day 7 after SAH in the double-injection model, the cerebral vasospasm was reversed in a dose-dependent manner: 2 × 10^–10^ mol/kg of CGRP reversed the vasospasm completely. The effect began to appear 5 min after the CGRP administration, continued for 4 h, and disappeared by 24 h. Of note, when the amounts of CGRP mentioned above were administered i.c., both mean arterial blood pressure and heart rate were only slightly increased and returned to the previous levels within several minutes.

In a similar study byImaizumi et al. (1996), experimental SAH was produced by i.c. injection of arterial blood in rabbits. The animals were treated with intrathecal administration of CGRP 3 days after SAH. The degree of vasospasm and the effect of CGRP were evaluated angiographically by measuring the basilar artery diameter. The basilar artery constricted to 73% of the pre-SAH values 3 days after SAH. Fifteen minutes after 10^–10^ mol/kg CGRP injection, the basilar artery dilated from 73 to 117% (*n* = 8), which was significantly larger than 67.1% in the vehicle group (*n* = 8; *p* < 0.01). At 6 h after 10^–10^ mol/kg CGRP injection, the basilar artery was still dilated to 90% (*p* < 0.05). In the 10^–11^ mol/kg CGRP group, the basilar artery was dilated to 87% (*p* < 0.05) 15 min after the injection. The injection of 10^–12^ mol/kg CGRP had no significant effect. The dilatory effect in the 10^–10^ mol/kg CGRP group was demonstrated up to 6 h after injection. Arterial blood pressure was stable after injection of CGRP.

Toshima et al. (1992) produced SAH in 41 rabbits by injecting i.c. autologous blood. The animals were randomly assigned to five groups and were sacrificed on day 2 post-SAH. Group 1 was the control group. Immediately prior to sacrifice, group 2 and 3 animals received a 2-h i.c. injection of vehicle or CGRP (100 ng/kg/min), respectively. Group 4 and 5 animals received a 2-h i.v. injection of vehicle or CGRP (100 ng/kg/min), respectively. The diameter of basilar artery in group 3 (i.c. CGRP) was significantly larger than that in group 2 (i.c. vehicle, *p* < 0.001). Similarly, the diameter of basilar artery in group 5 (i.v. CGRP) was significantly greater than that in group 4 (i.v. vehicle, *p *< 0.01). Although no significant difference was observed in mean arterial blood pressure between groups 2 and 3 (i.c. groups), there was a significant difference between i.v. groups 4 and 5 (lower in group 5, *p* < 0.01).

Ahmad et al. (1996) implanted a CGRP slow-release tablet i.c., containing either 24 or 153 µg of human αCGRP, 24 h after experimental SAH was induced in rabbits. Following implantation, the CGRP level in the CSF remained elevated for 5 days. The implantation of the tablet almost completely ameliorated angiographic vasospasm. Moreover, no significant systemic hypotension or neurological adverse event was associated with the treatment.

In a similar approach,Inoue et al. (1996) investigated the efficacy of a CGRP slow-release tablet for the prevention of cerebral vasospasm after SAH in monkeys. Experimental SAH was produced by the method ofEspinosa et al. (1984). The animal underwent a right frontotemporal craniectomy under sterile conditions. The dura mater was opened, and the arachnoid membrane was microsurgically incised until the ipsilateral internal carotid artery (ICA) and proximal portions of the MCA and anterior cerebral artery (ACA) were exposed. An autologous blood clot (1 ml/kg) was then placed around the exposed arteries to produce experimental SAH. For animals in the CGRP (*n* = 5) and placebo (*n* = 5) groups, a total of three tablets (total drug 1200 µg) were ipsilaterally placed under the frontal and temporal lobes at the time of SAH production. In the control group, cerebral vasospasm developed on day 7 (56% as an average of the ICA, MCA, and ACA). In the CGRP group, vasospasm was significantly ameliorated on average (75%, *p* < 0.02). The CGRP concentration in CSF was measurable only on day 7 for the CGRP group (6.5 nmol/L). No significant untoward reactions were recorded.

Toyoda et al. (2000) sought to determine whether adenovirus-mediated gene transfer *in vivo* of CGRP, ameliorates cerebral vasoconstriction after experimental SAH. Arterial blood was injected into the cisterna magna of rabbits to mimic SAH 5 days after injection of adenovirus or vehicle. After injection of adenovirus (*n* = 8), there was a 400-fold increase in CGRP in CSF. In rabbits treated with vehicle (controls, *n* = 8), basilar artery diameter after SAH was 25% smaller than before SAH (*p* < 0.0005). In rabbits treated with adenovirus, arterial diameter was similar before and after SAH. Furthermore, treatment of rabbits with adenovirus after experimental SAH prevented spasm of the basilar artery 2 days after SAH.

Likewise,Satoh et al. (2002) investigated whether a delayed treatment with adenovirus encoding CGRP gene, 2 days after experimental SAH, reduces cerebral vasospasm in a double-hemorrhage model (on days 0 and 2) of severe vasospasm in dogs. Severe vasospasm was observed in control SAH dogs (*n* = 12) on day 7, and the mean basilar artery diameter was 53% of baseline. In the group treated with adenovirus (*n* = 8), vasospasm was significantly reduced (the basilar artery diameter was 78% of baseline, *p* < 0.05 compared with the control SAH group). High levels of CGRP were measured in CSF from dogs that received adenovirus (115-fold greater than baseline levels).

Intracisternal gene transfer of CGRP was initially thought to be more useful than i.v. infusion, because the local gene transfer might avoid systemic effects of CGRP and achieve its sustained release into the central nervous system. However, there are several concerns, such as the inflammatory process induced by adenovirus, the difficulty in approaching the target cells in the presence of a large subarachnoid blood clot, and its potential ability for cancerous transformation of the affected cells.

### EFFECTS OF CGRP ADMINISTRATION ON CEREBRAL VASOSPASM AFTER SAH IN HUMANS (Table 2

**Table 2:** Studies of CGRP administration after aneurysmal SAH in humans.

Reference	**Sample size/patient type**	**Study design**	Way of CGRP administration	Dose	Primary outcome	Results	Adverse events
Juul etal. (1994)	5/ postoperative course after SAH	Not randomized, not controlled study	i.v. infusion	0.6 μg/min	H.I.	Significant reduction in H.I. during CGRP infusion compared to that before infusion	HR increase during CGRP infusion
Johnston etal. (1990)	15/ neurological deficit after surgical clipping of the aneurysm	Multicenter randomized placebo-controlled study	i.v. infusion	0.035 μg/min, doubled every 10 min, max 1.15 μg/min	Modified GCS	Of the nine patients who showed a treatment preference, eight favored CGRP (*p* < 0.05)	None
European CGRP in SAH study (1992)	117/ ischemic neurological deficit after operation for the aneurysm	Multicenter randomized placebo-controlled study	i.v. infusion	0.6 μg/min	Glasgow outcome scale	Relative risk of a bad outcome in CGRP-treated compared with controls 0.88 (95% CI: 0.6-1.26)	2/3 of the patients included in the CGRP group had reduced AP and did not complete treatment

*i.v., intravenous; AP, arterial pressure; HR, heart rate; SAH, subarachnoid hemorrhage; H.I., hemodynamic index; GCS, Glasgow coma scale.*

Juul et al. (1994) investigated the effect of i.v. CGRP infusion at a rate of 0.6 µg/min in five patients with vasoconstriction in the postoperative course after SAH, where the hemodynamic index (ratio between middle cerebral and ICA mean velocities) was used as an indicator of vasoconstriction. A significant reduction was found in the hemodynamic index during the CGRP infusion as compared to that before infusion (4.3 vs. 6.2, *p* < 0.05). However, no significant change was observed in pulsatility index (another indicator of vasospasm, equal to the difference between the systolic and diastolic flow velocities divided by the mean flow velocity), blood pressure, or consciousness during CGRP infusion. A significant increase in heart rate was observed during the infusion, while blood pressure remained unaltered.

Johnston et al. (1990) undertook a multicenter, randomized, placebo-controlled trial to study the safety and efficacy of i.v. CGRP treatment to reverse neurological deficits after surgical clipping of a ruptured intracranial aneurysm. Patients were enrolled if they had postoperative neurological deficit. Patients received CGRP or placebo in random order, 24 h apart. Fifteen patients were eventually included in the study. Infusion started at a rate sufficient to deliver 0.035 µg/min CGRP, and was doubled every 10 min until either a clinical response was obtained or a maximum dose of 1.15 µg/min was reached at 1 h. If the neurological deficit had not deteriorated and the patient had no side-effects by that time, the maximum infusion rate was continued for another 20 min. Regarding neurological changes according to the modified Glasgow Coma Scale, five patients did not improve on either treatment, one improved on both, eight improved on CGRP but not on placebo, and one improved on placebo but not on CGRP. Of the nine patients who showed a treatment preference, eight (88.9%) favored CGRP (*p* < 0.05). The mean duration of neurological improvement was 25 min, after which patients returned to their previous neurological status. There was a significant decrease in both systolic and diastolic blood pressures during the infusion of CGRP.

A larger, multicenter, randomized controlled trial (European CGRP in SAH study, 1992) investigated the effect of a postoperative infusion of CGRP on outcome at 3 months. Patients with aneurysmal SAH who underwent surgery entered the trial if an ischemic neurological deficit developed after the operation. A total of 117 patients entered the study (62 patients received CGRP and 55 standard management). The CGRP-treated patients received the drug by i.v. infusion at a rate of 0.6 µg/min. If systemic hypotension developed, the infusion rate was reduced to 0.45 µg/min, then to 0.3 µg/min, if the hypotension was still apparent. CGRP treatment was given for at least 4 h; patients who showed a satisfactory neurological response continued to receive treatment for up to 10 days (minimum of 4 days). The percentage of patients with a good outcome was slightly but not significantly higher in the CGRP than in the control group. The relative risk of a bad outcome in CGRP-treated compared with control patients was 0.88 (95% CI: 0.60–1.28). Interestingly, only a third of patients randomized to receive CGRP completed treatment, so two-thirds included in the treatment group for the analyses had limited exposure to CGRP, mainly due to arterial hypotension.

## CONCLUSION

The pathogenesis of vasospasm after SAH is complex, multifactorial, and incompletely understood. CGRP has shown promising results both *in vitro* and *in vivo*, mainly in animal models of experimental SAH. However, there is a lack of studies in humans. Systemic hypotension induced by the i.v. administration of the drug seems to be a serious problem. The encouraging results from the i.c. application of CGRP in animals could warrant large studies in humans with CGRP instillation into the subarachnoid space, in order to avoid hypotension and achieve even more efficient dilatation of the cerebral arteries.

## Conflict of Interest Statement

The authors declare that the research was conducted in the absence of any commercial or financial relationships that could be construed as a potential conflict of interest.

## References

[B1] Ahmad I. Imaizumi S. Shimizu H. Kaminuma T. Ochiai N. Tajima M. (1996). Development of calcitonin gene-related peptide slow-release tablet implanted in CSF space for prevention of cerebral vasospasm after experimental subarachnoid haemorrhage. *Acta Neurochir. (Wien)* 138 1230–1240 8955444 10.1007/BF01809753

[B2] Aiyar N. Disa J. Stadel J. M. Lysko P. G. (1999). Calcitonin gene-related peptide receptor independently stimulates 3′,5′-cyclic adenosine monophosphate and Ca^2+^ signaling pathways. *Mol. Cell. Biochem.* 197 179–185 10485337 10.1023/a:1006962221332

[B3] Al-Tamimi Y. Z. Orsi N. M. Quinn A. C. Homer-Vanniasinkam S. Ross S. A. (2010). A review of delayed ischemic neurologic deficit following aneurysmal subarachnoid hemorrhage: historical overview, current treatment, and pathophysiology. *World Neurosurg.* 73 654–667 20934153 10.1016/j.wneu.2010.02.005

[B4] Alevizaki M. Shiraishi A. Rassool F. V. Ferrier G. J. MacIntyre I. Legon S. (1986). The calcitonin-like sequence of the beta CGRP gene. *FEBS Lett.* 206 47–52 3489641 10.1016/0014-5793(86)81338-2

[B5] Amara S. G. Jonas V. Rosenfeld M. G. Ong E. S. Evans R. M. (1982). Alternative RNA processing in calcitonin gene expression generates mRNAs encoding different polypeptide products. *Nature* 298 240–244 6283379 10.1038/298240a0

[B6] Amin-Hanjani S. Ogilvy C. S. Barker F. G. (2004). Does intracisternal thrombolysis prevent vasospasm after aneurysmal subarachnoid hemorrhage? A meta-analysis. *Neurosurgery* 54 326–334 14744278 10.1227/01.neu.0000103488.94855.4f

[B7] Arienta C. Balbi S. Caroli M. Fumagalli G. (1991). Depletion of calcitonin gene-related peptide in perivascular nerves during acute phase of posthemorrhagic vasospasm in the rabbit. *Brain Res. Bull.* 27 605–609 1756379 10.1016/0361-9230(91)90034-h

[B8] Asano T. Matsui T. (1999). Antioxidant therapy against cerebral vasospasm following aneurysmal subarachnoid hemorrhage. *Cell Mol. Neurobiol.* 19 31–44 10079963 10.1023/A:1006908422937 PMC11545434

[B9] Benarroch E. E. (2011). CGRP: sensory neuropeptide with multiple neurologic implications. *Neurology* 77 281–287 21768598 10.1212/WNL.0b013e31822550e2

[B10] Brain S. D. Williams T. J. (1988). Substance P regulates the vasodilator activity of calcitonin gene-related peptide. *Nature* 335 73–75 2457810 10.1038/335073a0

[B11] Brain S. D. Williams T. J. (1989). Interactions between the tachykinins and calcitonin gene-related peptide lead to the modulation of oedema formation and blood flow in rat skin. *Br. J. Pharmacol.* 97 77–82 2470460 10.1111/j.1476-5381.1989.tb11926.x PMC1854478

[B12] Brain S. D. Williams T. J. Tippins J. R. Morris H. R. MacIntyre I. (1985). Calcitonin gene-related peptide is a potent vasodilator. *Nature* 313 54–56 3917554 10.1038/313054a0

[B13] Breeze A. L. Harvey T. S. Bazzo R. Campbell I. D. (1991). Solution structure of human calcitonin gene-related peptide by 1H NMR and distance geometry with restrained molecular dynamics. *Biochemistry* 30 575–582 1988044 10.1021/bi00216a036

[B14] Brisman J. L. Eskridge J. M. Newell D. W. (2006). Neurointerventional treatment of vasospasm. *Neurol. Res.* 28 769–776 17164040 10.1179/016164106X152043

[B15] Butt E. Bernhardt M. Smolenski A. Kotsonis P. Frohlich L. G. Sickmann A. (2000). Endothelial nitric-oxide synthase (type III) is activated and becomes calcium independent upon phosphorylation by cyclic nucleotide-dependent protein kinases. *J. Biol. Chem.* 275 5179–5187 10671564 10.1074/jbc.275.7.5179

[B16] Carpenter K. A. Schmidt R. von Mentzer B. Haglund U. Roberts E. Walpole C. (2001). Turn structures in CGRP C-terminal analogues promote stable arrangements of key residue side chains. *Biochemistry* 40 8317–8325 11444978 10.1021/bi0102860

[B17] Chiba T. Yamaguchi A. Yamatani T. Nakamura A. Morishita T. Inui T. (1989). Calcitonin gene-related peptide receptor antagonist human CGRP-(8–37). *Am. J. Physiol.* 256 E331–E335 2537579 10.1152/ajpendo.1989.256.2.E331

[B18] Chow M. Dumont A. S. Kassell N. F. (2002). Endothelin receptor antagonists and cerebral vasospasm: an update. *Neurosurgery* 51 1333–1341 12445337

[B19] Clark J. F. Sharp F. R. (2006). Bilirubin oxidation products (BOXes) and their role in cerebral vasospasm after subarachnoid hemorrhage. *J. Cereb. Blood Flow Metab.* 26 1223–1233 16467784 10.1038/sj.jcbfm.9600280

[B20] Conner A. C. Hay D. L. Howitt S. G. Kilk K. Langel U. Wheatley M. (2002). Interaction of calcitonin-gene-related peptide with its receptors. *Biochem. Soc. Trans.* 30 451–455 12196113 10.1042/bst0300451

[B21] Conner A. C. Hay D. L. Simms J. Howitt S. G. Schindler M. Smith D. M. (2005). A key role for transmembrane prolines in calcitonin receptor-like receptor agonist binding and signalling: implications for family B G-protein-coupled receptors. *Mol. Pharmacol.* 67 20–31 15615699

[B22] Conner A. C. Simms J. Barwell J. Wheatley M. Poyner D. R. (2007). Ligand binding and activation of the CGRP receptor. *Biochem. Soc. Trans.* 35 729–732 17635135 10.1042/BST0350729

[B23] Crossman D. C. Dashwood M. R. Brain S. D. McEwan J. Pearson J. D. (1990). Action of calcitonin gene-related peptide upon bovine vascular endothelial and smooth muscle cells grown in isolation and co-culture. *Br. J. Pharmacol.* 99 71–76 2184911 10.1111/j.1476-5381.1990.tb14656.x PMC1917523

[B24] Dennis T. Fournier A. Cadieux A. Pomerleau F. Jolicoeur F. B. St Pierre S. (1990). hCGRP837, a calcitonin gene-related peptide antagonist revealing calcitonin gene-related peptide receptor heterogeneity in brain and periphery. *J. Pharmacol. Exp. Ther.* 254 123–128 2164085

[B25] Dennis T. Fournier A. St Pierre S. Quirion R. (1989). Structure-activity profile of calcitonin gene-related peptide in peripheral and brain tissues. Evidence for receptor multiplicity. *J. Pharmacol. Exp. Ther.* 251 718–725 2553933

[B26] Disa J. Parameswaran N. Nambi P. Aiyar N. (2000). Involvement of cAMP-dependent protein kinase and pertussis toxin-sensitive G-proteins in CGRP mediated JNK activation in human neuroblastoma cell line. *Neuropeptides* 34 229–233 11021985 10.1054/npep.2000.0810

[B27] Dorhout Mees S. M. Algra A. Vandertop W. P. van Kooten F. Kuijsten H. A. Boiten J. (2012). Magnesium for aneurysmal subarachnoid haemorrhage (MASH-2): a randomised placebo-controlled trial. *Lancet* 380 44–49 22633825 10.1016/S0140-6736(12)60724-7 PMC3391717

[B28] Dorsch N. W. (1995). Cerebral arterial spasm – a clinical review. *Br. J. Neurosurg.* 9 403–412 7546361 10.1080/02688699550041403

[B29] Dreier J. P. Major S. Manning A. Woitzik J. Drenckhahn C. Steinbrink J. (2009). Cortical spreading ischaemia is a novel process involved in ischaemic damage in patients with aneurysmal subarachnoid haemorrhage. *Brain* 132 1866–1881 19420089 10.1093/brain/awp102 PMC2702835

[B30] Duerrschmidt N. Wippich N. Goettsch W. Broemme H. J. Morawietz H. (2000). Endothelin-1 induces NAD(P)H oxidase in human endothelial cells. *Biochem. Biophys. Res. Commun.* 269 713–717 10720482 10.1006/bbrc.2000.2354

[B31] Dumont A. S. Dumont R. J. Chow M. M. Lin C. L. Calisaneller T. Ley K. F. (2003). Cerebral vasospasm after subarachnoid hemorrhage: putative role of inflammation. *Neurosurgery* 53 123–133 12823881 10.1227/01.neu.0000068863.37133.9e

[B32] Dumont Y. Fournier A. St-Pierre S. Quirion R. (1997). A potent and selective CGRP2 agonist, [Cys(Et)2,7]hCGRP alpha: comparison in prototypical CGRP1 and CGRP2 in vitro bioassays. *Can. J. Physiol. Pharmacol.* 75 671–676 9276147

[B33] Edvinsson L. Delgado-Zygmunt T. Ekman R. Jansen I. Svendgaard N. A. Uddman R. (1990). Involvement of perivascular sensory fibers in the pathophysiology of cerebral vasospasm following subarachnoid hemorrhage. *J. Cereb. Blood Flow Metab.* 10 602–607 1696581 10.1038/jcbfm.1990.111

[B34] Edvinsson L. Ekman R. Jansen I. McCulloch J. Mortensen A. Uddman R. (1991). Reduced levels of calcitonin gene-related peptide-like immunoreactivity in human brain vessels after subarachnoid haemorrhage. *Neurosci. Lett.* 121 151–154 2020372 10.1016/0304-3940(91)90672-g

[B35] Edvinsson L. Fredholm B. B. Hamel E. Jansen I. Verrecchia C. (1985). Perivascular peptides relax cerebral arteries concomitant with stimulation of cyclic adenosine monophosphate accumulation or release of an endothelium-derived relaxing factor in the cat. *Neurosci. Lett.* 58 213–217 2995876 10.1016/0304-3940(85)90166-1

[B36] Edvinsson L. Gulbenkian S. Barroso C. P. Cunha e Sa M. Polak J. M. Mortensen A. (1998). Innervation of the human middle meningeal artery: immunohistochemistry, ultrastructure, and role of endothelium for vasomotility. *Peptides* 19 1213–1225 9786171 10.1016/s0196-9781(98)00066-7

[B37] Egge A. Waterloo K. Sjoholm H. Solberg T. Ingebrigtsen T. Romner B. (2001). Prophylactic hyperdynamic postoperative fluid therapy after aneurysmal subarachnoid hemorrhage: a clinical, prospective, randomized, controlled study. *Neurosurgery* 49 593–605 11523669 10.1097/00006123-200109000-00012

[B38] Espinosa F. Weir B. Overton T. Castor W. Grace M. Boisvert D. (1984). A randomized placebo-controlled double-blind trial of nimodipine after SAH in monkeys. Part 1: clinical and radiological findings. *J. Neurosurg.* 60 1167–1175 6726360 10.3171/jns.1984.60.6.1167

[B39] European CGRP in Subarachnoid Haemorrhage Study Group. (1992). Effect of calcitonin-gene-related peptide in patients with delayed postoperative cerebral ischaemia after aneurysmal subarachnoid haemorrhage. European CGRP in Subarachnoid Haemorrhage Study Group. *Lancet* 339 831–834 1347857

[B40] Evans B. N. Rosenblatt M. I. Mnayer L. O. Oliver K. R. Dickerson I. M. (2000). CGRP-RCP, a novel protein required for signal transduction at calcitonin gene-related peptide and adrenomedullin receptors. *J. Biol. Chem.* 275 31438–31443 10903324 10.1074/jbc.M005604200

[B41] Fassbender K. Hodapp B. Rossol S. Bertsch T. Schmeck J. Schutt S. (2001). Inflammatory cytokines in subarachnoid haemorrhage: association with abnormal blood flow velocities in basal cerebral arteries. *J. Neurol. Neurosurg. Psychiatry* 70 534–537 11254783 10.1136/jnnp.70.4.534 PMC1737308

[B42] Fassbender K. Hodapp B. Rossol S. Bertsch T. Schmeck J. Schutt S. (2000). Endothelin-1 in subarachnoid hemorrhage: an acute-phase reactant produced by cerebrospinal fluid leukocytes. *Stroke* 31 2971–2975 11108758 10.1161/01.str.31.12.2971

[B43] Fernandez-Patron C. Stewart K. G. Zhang Y. Koivunen E. Radomski M. W. Davidge S. T. (2000). Vascular matrix metalloproteinase-2-dependent cleavage of calcitonin gene-related peptide promotes vasoconstriction. *Circ. Res.* 87 670–676 11029402 10.1161/01.res.87.8.670

[B44] Ferro A. Queen L. R. Priest R. M. Xu B. Ritter J. M. Poston L. (1999). Activation of nitric oxide synthase by beta 2-adrenoceptors in human umbilical vein endothelium *in vitro*. *Br. J. Pharmacol.* 126 1872–1880 10372832 10.1038/sj.bjp.0702512 PMC1565965

[B45] Freeland K. Liu Y. Z. Latchman D. S. (2000). Distinct signalling pathways mediate the cAMP response element (CRE)-dependent activation of the calcitonin gene-related peptide gene promoter by cAMP and nerve growth factor. *Biochem. J.* 345(Pt 2) 233–238 10620499 PMC1220751

[B46] Frontera J. A. Fernandez A. Schmidt J. M. Claassen J. Wartenberg K. E. Badjatia N. (2009). Defining vasospasm after subarachnoid hemorrhage: what is the most clinically relevant definition? *Stroke* 40 1963–1968 19359629 10.1161/STROKEAHA.108.544700

[B47] Goretski J. Hollocher T. C. (1988). Trapping of nitric oxide produced during denitrification by extracellular hemoglobin. *J. Biol. Chem.* 263 2316–2323 3339013

[B48] Gray D. W. Marshall I. (1992a). Human alpha-calcitonin gene-related peptide stimulates adenylate cyclase and guanylate cyclase and relaxes rat thoracic aorta by releasing nitric oxide. *Br. J. Pharmacol.* 107 691–696 1361870 10.1111/j.1476-5381.1992.tb14508.x PMC1907745

[B49] Gray D. W. Marshall I. (1992b). Nitric oxide synthesis inhibitors attenuate calcitonin gene-related peptide endothelium-dependent vasorelaxation in rat aorta. *Eur. J. Pharmacol.* 212 37–42 1555637 10.1016/0014-2999(92)90069-g

[B50] Grisham M. B. Granger D. N. Lefer D. J. (1998). Modulation of leukocyte-endothelial interactions by reactive metabolites of oxygen and nitrogen: relevance to ischemic heart disease. *Free Radic. Biol. Med.* 25 404–433 9741579 10.1016/s0891-5849(98)00094-x

[B51] Gulbenkian S. Saetrum O. O. Ekman R. Costa A. N. Wharton J. Polak J. M. (1993). Peptidergic innervation of human epicardial coronary arteries. *Circ. Res.* 73 579–588 7688669 10.1161/01.res.73.3.579

[B52] Haley E. C. Jr. Kassell N. F. Apperson-Hansen C. Maile M. H. Alves W. M. (1997). A randomized, double-blind, vehicle-controlled trial of tirilazad mesylate in patients with aneurysmal subarachnoid hemorrhage: a cooperative study in North America. *J. Neurosurg.* 86 467–474 9046304 10.3171/jns.1997.86.3.0467

[B53] Hay D. L. (2007). What makes a CGRP2 receptor? *Clin. Exp. Pharmacol. Physiol.* 34 963–971 17714080 10.1111/j.1440-1681.2007.04703.x

[B54] Hino A. Tokuyama Y. Weir B. Takeda J. Yano H. Bell G. I. (1996). Changes in endothelial nitric oxide synthase mRNA during vasospasm after subarachnoid hemorrhage in monkeys. *Neurosurgery* 39 562–567 8875487 10.1097/00006123-199609000-00026

[B55] Hirashima Y. Nakamura S. Endo S. Kuwayama N. Naruse Y. Takaku A. (1997). Elevation of platelet activating factor, inflammatory cytokines, and coagulation factors in the internal jugular vein of patients with subarachnoid hemorrhage. *Neurochem. Res.* 22 1249–1255 9342729 10.1023/a:1021985030331

[B56] Hoare S. R. (2005). Mechanisms of peptide and nonpeptide ligand binding to Class B G-protein-coupled receptors. *Drug Discov. Today* 10 417–427 15808821 10.1016/S1359-6446(05)03370-2

[B57] Holzer P. (2000). Local microcirculatory reflexes and afferent signalling in response to gastric acid challenge. *Gut* 47(Suppl. 4) iv46–iv48 11076911 10.1136/gut.47.suppl_4.iv46 PMC1766803

[B58] Horowitz A. Menice C. B. Laporte R. Morgan K. G. (1996). Mechanisms of smooth muscle contraction. *Physiol. Rev.* 76 967–1003 8874491 10.1152/physrev.1996.76.4.967

[B59] Huang J van Gelder J. M. (2002). The probability of sudden death from rupture of intracranial aneurysms: a meta-analysis. *Neurosurgery* 51 1101–1105 12383354 10.1097/00006123-200211000-00001

[B60] Ignarro L. J. (1990). Biosynthesis and metabolism of endothelium-derived nitric oxide. *Annu. Rev. Pharmacol. Toxicol.* 30 535–560 2188578 10.1146/annurev.pa.30.040190.002535

[B61] Imaizumi S. Shimizu H. Ahmad I. Kaminuma T. Tajima M. Yoshimoto T. (1996). Effect of calcitonin gene-related peptide on delayed cerebral vasospasm after experimental subarachnoid hemorrhage in rabbits. *Surg. Neurol.* 46 263–270 8781597 10.1016/0090-3019(96)00048-1

[B62] Ingall T. Asplund K. Mahonen M. Bonita R. (2000). A multinational comparison of subarachnoid hemorrhage epidemiology in the WHO MONICA stroke study. *Stroke* 31 1054–1061 10797165 10.1161/01.str.31.5.1054

[B63] Inoue T. Shimizu H. Kaminuma T. Tajima M. Watabe K. Yoshimoto T. (1996). Prevention of cerebral vasospasm by calcitonin gene-related peptide slow-release tablet after subarachnoid hemorrhage in monkeys. *Neurosurgery* 39 984–990 8905755 10.1097/00006123-199611000-00020

[B64] Ishiguro M. Murakami K. Link T. Zvarova K. Tranmer B. I. Morielli A. D. (2008). Acute and chronic effects of oxyhemoglobin on voltage-dependent ion channels in cerebral arteries. *Acta Neurochir. Suppl.* 104 99–102 18456998 10.1007/978-3-211-75718-5_19

[B65] Ishiguro M. Wellman T. L. Honda A. Russell S. R. Tranmer B. I. Wellman G. C. (2005). Emergence of a R-type Ca^2+^ channel (CaV 2.3) contributes to cerebral artery constriction after subarachnoid hemorrhage. * Circ. Res.* 96 419–426 15692089 10.1161/01.RES.0000157670.49936.da

[B66] Jestaedt L. Pham M. Bartsch A. J. Kunze E. Roosen K. Solymosi L. (2008). The impact of balloon angioplasty on the evolution of vasospasm-related infarction after aneurysmal subarachnoid hemorrhage. *Neurosurgery* 62 610–617 18301345 10.1227/01.NEU.0000311351.32904.8B

[B67] Johnston F. G. Bell B. A. Robertson I. J. Miller J. D. Haliburn C. O’Shaughnessy D. (1990). Effect of calcitonin-gene-related peptide on postoperative neurological deficits after subarachnoid haemorrhage. *Lancet* 335 869–872 1969982 10.1016/0140-6736(90)90473-i

[B68] Jung C. S. Iuliano B. A. Harvey-White J. Espey M. G. Oldfield E. H. Pluta R. M. (2004). Association between cerebrospinal fluid levels of asymmetric dimethyl-L-arginine, an endogenous inhibitor of endothelial nitric oxide synthase, and cerebral vasospasm in a primate model of subarachnoid hemorrhage. *J. Neurosurg.* 101 836–842 15543672 10.3171/jns.2004.101.5.0836

[B69] Juul R. Aakhus S. Bjornstad K. Gisvold S. E. Brubakk A. O. Edvinsson L. (1994). Calcitonin gene-related peptide (human alpha-CGRP) counteracts vasoconstriction in human subarachnoid haemorrhage. *Neurosci. Lett.* 170 67–70 8041516 10.1016/0304-3940(94)90240-2

[B70] Juul R. Edvinsson L. Gisvold S. E. Ekman R. Brubakk A. O. Fredriksen T. A. (1990). Calcitonin gene-related peptide-LI in subarachnoid haemorrhage in man. Signs of activation of the trigemino-cerebrovascular system? *Br. J. Neurosurg.* 4 171–179 2204353 10.3109/02688699008992720

[B71] Juul R. Hara H. Gisvold S. E. Brubakk A. O. Fredriksen T. A. Waldemar G. (1995). Alterations in perivascular dilatory neuropeptides (CGRP, SP, VIP) in the external jugular vein and in the cerebrospinal fluid following subarachnoid haemorrhage in man. *Acta Neurochir. (Wien)* 132 32–41 7538726 10.1007/BF01404845

[B72] Juvela S. (2000). Plasma endothelin concentrations after aneurysmal subarachnoid hemorrhage. *J. Neurosurg.* 92 390–400 10701524 10.3171/jns.2000.92.3.0390

[B73] Kassell N. F. Peerless S. J. Durward Q. J. Beck D. W. Drake C. G. Adams H. P. (1982). Treatment of ischemic deficits from vasospasm with intravascular volume expansion and induced arterial hypertension. *Neurosurgery* 11 337–343 7133349 10.1227/00006123-198209000-00001

[B74] Kasuya H. Onda H. Sasahara A. Takeshita M. Hori T. (2005). Application of nicardipine prolonged-release implants: analysis of 97 consecutive patients with acute subarachnoid hemorrhage. *Neurosurgery* 56 895–902 15854236

[B75] Kim Y. W. Lawson M. F. Hoh B. L. (2012). Nonaneurysmal subarachnoid hemorrhage: an update. *Curr. Atheroscler. Rep*. 14 328–334 22638876 10.1007/s11883-012-0256-x

[B76] Koide M. Nystoriak M. A. Brayden J. E. Wellman G. C. (2011). Impact of subarachnoid hemorrhage on local and global calcium signaling in cerebral artery myocytes. *Acta Neurochir. Suppl.* 110(Pt 1) 145–150 21116930 10.1007/978-3-7091-0353-1_25 PMC3057755

[B77] Kosnik E. J. Hunt W. E. (1976). Postoperative hypertension in the management of patients with intracranial arterial aneurysms. *J. Neurosurg.* 45 148–154 939973 10.3171/jns.1976.45.2.0148

[B78] Kostron H. Twerdy K. Grunert V. (1988). The calcium entry blocker nimodipine improves the quality of life of patients operated on for cerebral aneurysms. A 5-year follow-up analysis. *Neurochirurgia (Stuttg.)* 31 150–153 3231282 10.1055/s-2008-1053923

[B79] Kraenzlin M. E. Ch’ng J. L. Mulderry P. K. Ghatei M. A. Bloom S. R. (1985). Infusion of a novel peptide, calcitonin gene-related peptide (CGRP) in man. Pharmacokinetics and effects on gastric acid secretion and on gastrointestinal hormones. *Regul. Pept.* 10 189–197 3922013 10.1016/0167-0115(85)90013-8

[B80] Kwan A. L. Lin C. L. Chang C. Z. Winardi D. Yen C. P. Wu S. C. (2002). Oral administration of an inhibitor of endothelin-converting enzyme attenuates cerebral vasospasm following experimental subarachnoid haemorrhage in rabbits. *Clin. Sci. (Lond.)* 103(Suppl. 48) 414S–417S 12193135 10.1042/CS103S414S

[B81] Laufer R. Changeux J. P. (1989). Calcitonin gene-related peptide and cyclic AMP stimulate phosphoinositide turnover in skeletal muscle cells. Interaction between two second messenger systems. *J. Biol. Chem.* 264 2683–2689 2536720

[B82] Le Greves P. Nyberg F. Hokfelt T. Terenius L. (1989). Calcitonin gene-related peptide is metabolized by an endopeptidase hydrolyzing substance P. *Regul. Pept.* 25 277–286 2475892 10.1016/0167-0115(89)90176-6

[B83] Lennihan L. Mayer S. A. Fink M. E. Beckford A. Paik M. C. Zhang H. (2000). Effect of hypervolemic therapy on cerebral blood flow after subarachnoid hemorrhage: a randomized controlled trial. *Stroke* 31 383–391 10657410 10.1161/01.str.31.2.383

[B84] Lin C. L. Hsu Y. T. Lin T. K. Morrow J. D. Hsu J. C. Hsu. Y. H., et al. (2006). Increased levels of F2-isoprostanes following aneurysmal subarachnoid hemorrhage in humans. *Free Radic. Biol. Med.* 40 1466–1473 16631536 10.1016/j.freeradbiomed.2005.12.019

[B85] Lynch J. R. Wang H. McGirt M. J. Floyd J. Friedman A. H. Coon A. L. (2005). Simvastatin reduces vasospasm after aneurysmal subarachnoid hemorrhage: results of a pilot randomized clinical trial. *Stroke* 36 2024–2026 16051891 10.1161/01.STR.0000177879.11607.10

[B86] Macdonald R. L. Higashida R. T. Keller E. Mayer S. A. Molyneux A. Raabe A. (2011). Clazosentan, an endothelin receptor antagonist, in patients with aneurysmal subarachnoid haemorrhage undergoing surgical clipping: a randomised, double-blind, placebo-controlled phase 3 trial (CONSCIOUS-2). *Lancet Neurol.* 10 618–625 21640651 10.1016/S1474-4422(11)70108-9

[B87] Macdonald R. L. Kassell N. F. Mayer S. Ruefenacht D. Schmiedek P. Weidauer S. (2008). Clazosentan to overcome neurological ischemia and infarction occurring after subarachnoid hemorrhage (CONSCIOUS-1): randomized, double-blind, placebo-controlled phase 2 dose-finding trial. *Stroke* 39 3015–3021 18688013 10.1161/STROKEAHA.108.519942

[B88] Macdonald R. L. Weir B. K. Runzer T. D. Grace M. G. Findlay J. M. Saito K. (1991). Etiology of cerebral vasospasm in primates. *J. Neurosurg.* 75 415–424 1869943 10.3171/jns.1991.75.3.0415

[B89] Maggi C. A. Rovero P. Giuliani S. Evangelista S. Regoli D. Meli A. (1990). Biological activity of N-terminal fragments of calcitonin gene-related peptide. *Eur. J. Pharmacol.* 179 217–219 2364983 10.1016/0014-2999(90)90422-3

[B90] Mayberg M. R. Okada T. Bark D. H. (1990). The role of hemoglobin in arterial narrowing after subarachnoid hemorrhage. *J. Neurosurg.* 72 634–640 2319322 10.3171/jns.1990.72.4.0634

[B91] McCulloch J. Uddman R. Kingman T. A. Edvinsson L. (1986). Calcitonin gene-related peptide: functional role in cerebrovascular regulation. *Proc. Natl. Acad. Sci. U.S.A.* 83 5731–5735 3488550 10.1073/pnas.83.15.5731 PMC386363

[B92] McLatchie L. M. Fraser N. J. Main M. J. Wise A. Brown J. Thompson N. (1998). RAMPs regulate the transport and ligand specificity of the calcitonin-receptor-like receptor. *Nature* 393 333–339 9620797 10.1038/30666

[B93] Mee E. Dorrance D. Lowe D. Neil-Dwyer G. (1988). Controlled study of nimodipine in aneurysm patients treated early after subarachnoid hemorrhage. *Neurosurgery* 22 484–491 3283595 10.1227/00006123-198803000-00006

[B94] Meens M. J. Compeer M. G. Hackeng T. M. van Zandvoort M. A. Janssen B. J De Mey J. G. (2010). Stimuli of sensory-motor nerves terminate arterial contractile effects of endothelin-1 by CGRP and dissociation of ET-1/ET(A)-receptor complexes. *PLoS ONE* 5 e10917 10.1371/journal.pone.0010917 PMC2879375 20532232

[B95] Meens M. J. Mattheij N. J. van Loenen P. B. Spijkers L. J. Lemkens P. Nelissen. J, et al. (2012). G-protein betagamma subunits in vasorelaxing and anti-endothelinergic effects of calcitonin gene-related peptide. *Br. J. Pharmacol.* 166 297–308 22074193 10.1111/j.1476-5381.2011.01774.x PMC3415655

[B96] Mulderry P. K. Ghatei M. A. Spokes R. A. Jones P. M. Pierson A. M. Hamid Q. A. (1988). Differential expression of alpha-CGRP and beta-CGRP by primary sensory neurons and enteric autonomic neurons of the rat. *Neuroscience* 25 195–205 2839796 10.1016/0306-4522(88)90018-8

[B97] Neil-Dwyer G. Mee E. Dorrance D. Lowe D. (1987). Early intervention with nimodipine in subarachnoid haemorrhage. *Eur. Heart J.* 8(Suppl. K) 41–47 3450521 10.1093/eurheartj/8.suppl_k.41

[B98] Nelson M. T. Huang Y. Brayden J. E. Hescheler J. Standen N. B. (1990). Arterial dilations in response to calcitonin gene-related peptide involve activation of K^+^ channels. *Nature* 344 770–773 2109832 10.1038/344770a0

[B99] Nozaki K. Kikuchi H. Mizuno N. (1989a). Changes of calcitonin gene-related peptide-like immunoreactivity in cerebrovascular nerve fibers in the dog after experimentally produced subarachnoid hemorrhage. *Neurosci. Lett.* 102 27–32 2789350 10.1016/0304-3940(89)90302-9

[B100] Nozaki K. Uemura Y. Okamoto S. Kikuchi H. Mizuno N. (1989b). Relaxant effect of calcitonin gene-related peptide on cerebral arterial spasm induced by experimental subarachnoid hemorrhage in dogs. *J. Neurosurg.* 71 558–564 2795174 10.3171/jns.1989.71.4.0558

[B101] Ohkuma H. Fujita S. Suzuki S. (2002). Incidence of aneurysmal subarachnoid hemorrhage in Shimokita, Japan, from 1989 to 1998. *Stroke* 33 195–199 11779910 10.1161/hs0102.101891

[B102] Ohkuma H. Tsurutani H. Suzuki S. (2001). Incidence and significance of early aneurysmal rebleeding before neurosurgical or neurological management. *Stroke* 32 1176–1180 11340229 10.1161/01.str.32.5.1176

[B103] Padilla B. E. Cottrell G. S. Roosterman D. Pikios S. Muller L. Steinhoff, M., et al. (2007). Endo-thelin-converting enzyme-1 regulates endosomal sorting of calcitonin receptor-like receptor and beta-arrestins. *J. Cell Biol.* 179 981–997 18039931 10.1083/jcb.200704053 PMC2099187

[B104] Petermann J. B. Born W. Chang J. Y. Fischer J. A. (1987). Identification in the human central nervous system, pituitary, and thyroid of a novel calcitonin gene-related peptide, and partial amino acid sequence in the spinal cord. *J. Biol. Chem.* 262 542–545 3492492

[B105] Pierce K. L. Premont R. T. Lefkowitz R. J. (2002). Seven-transmembrane receptors. *Nat. Rev. Mol. Cell Biol.* 3 639–650 12209124 10.1038/nrm908

[B106] Pluta R. M. (2005). Delayed cerebral vasospasm and nitric oxide: review, new hypothesis, and proposed treatment. *Pharmacol. Ther.* 105 23–56 15626454 10.1016/j.pharmthera.2004.10.002

[B107] Pluta R. M. Dejam A. Grimes G. Gladwin M. T. Oldfield E. H. (2005). Nitrite infusions to prevent delayed cerebral vasospasm in a primate model of subarachnoid hemorrhage. *JAMA* 293 1477–1484 15784871 10.1001/jama.293.12.1477

[B108] Pluta R. M. Thompson B. G. Dawson T. M. Snyder S. H. Boock R. J. Oldfield E. H. (1996). Loss of nitric oxide synthase immunoreactivity in cerebral vasospasm. *J. Neurosurg.* 84 648–654 8613858 10.3171/jns.1996.84.4.0648

[B109] Polin R. S. Bavbek M. Shaffrey M. E. Billups K. Bogaev C. A. Kassell N. F. (1998). Detection of soluble E-selectin, ICAM-1, VCAM-1, and L-selectin in the cerebrospinal fluid of patients after subarachnoid hemorrhage. *J. Neurosurg.* 89 559–567 9761049 10.3171/jns.1998.89.4.0559

[B110] Poyner D. (1995). Pharmacology of receptors for calcitonin gene-related peptide and amylin. *Trends Pharmacol. Sci.* 16 424–428 8578616 10.1016/s0165-6147(00)89093-8

[B111] Poyner D. R. Sexton P. M. Marshall I. Smith D. M. Quirion R. Born W. (2002). International Union of Pharmacology. XXXII. The mammalian calcitonin gene-related peptides, adrenomedullin, amylin, and calcitonin receptors. *Pharmacol. Rev.* 54 233–246 12037140 10.1124/pr.54.2.233

[B112] Queen L. R. Xu B. Horinouchi K. Fisher I. Ferro A. (2000). beta(2)-adrenoceptors activate nitric oxide synthase in human platelets. *Circ. Res.* 87 39–44 10884370 10.1161/01.res.87.1.39

[B113] Quirion R. Van Rossum D. Dumont Y. St-Pierre S. Fournier A. (1992). Characterization of CGRP1 and CGRP2 receptor subtypes. *Ann. N. Y. Acad. Sci.* 657 88–105 1322107 10.1111/j.1749-6632.1992.tb22759.x

[B114] Sams-Nielsen A. Orskov C. Jansen-Olesen I. (2001). Pharmacological evidence for CGRP uptake into perivascular capsaicin sensitive nerve terminals. *Br. J. Pharmacol.* 132 1145–1153 11226146 10.1038/sj.bjp.0703910 PMC1572649

[B115] Santhanam A. V. Smith L. A. Akiyama M. Rosales A. G. Bailey K. R. Katusic Z. S. (2005). Role of endothelial NO synthase phosphorylation in cerebrovascular protective effect of recombinant erythropoietin during subarachnoid hemorrhage-induced cerebral vasospasm. *Stroke* 36 2731–2737 16269632 10.1161/01.STR.0000190021.85035.5b

[B116] Satoh M. Perkins E. Kimura H. Tang J. Chun Y. Heistad D. D. (2002). Posttreatment with adenovirus-mediated gene transfer of calcitonin gene-related peptide to reverse cerebral vasospasm in dogs. *J. Neurosurg.* 97 136–142 12134904 10.3171/jns.2002.97.1.0136

[B117] Seifert V. Loffler B. M. Zimmermann M. Roux S. Stolke D. (1995). Endothelin concentrations in patients with aneurysmal subarachnoid hemorrhage. Correlation with cerebral vasospasm, delayed ischemic neurological deficits, and volume of hematoma. *J. Neurosurg.* 82 55–62 7815135 10.3171/jns.1995.82.1.0055

[B118] Sexton P. M. Morfis M. Tilakaratne N. Hay D. L. Udawela M. Christopoulos G. (2006). Complexing receptor pharmacology: modulation of family B G protein-coupled receptor function by RAMPs. *Ann. N. Y. Acad. Sci.* 1070 90–104 16888151 10.1196/annals.1317.076

[B119] Sexton P. M. Poyner D. R. Simms J. Christopoulos A. Hay D. L. (2009). Modulating receptor function through RAMPs: can they represent drug targets in themselves? *Drug Discov. Today* 14 413–419 19150656 10.1016/j.drudis.2008.12.009

[B120] Shibuya M. Suzuki Y. Sugita K. Saito I. Sasaki T. Takakura K. (1992). Effect of AT877 on cerebral vasospasm after aneurysmal subarachnoid hemorrhage. Results of a prospective placebo-controlled double-blind trial. *J. Neurosurg.* 76 571–577 1545249 10.3171/jns.1992.76.4.0571

[B121] Steenbergh P. H. Hoppener J. W. Zandberg J. Lips C. J. Jansz H. S. (1985). A second human calcitonin/CGRP gene. *FEBS Lett.* 183 403–407 2985435 10.1016/0014-5793(85)80820-6

[B122] Steenbergh P. H. Hoppener J. W. Zandberg J. Visser A. Lips C. J. Jansz H. S. (1986). Structure and expression of the human calcitonin/CGRP genes. *FEBS Lett.* 209 97–103 3492393 10.1016/0014-5793(86)81091-2

[B123] Struthers A. D. Brown M. J. Macdonald D. W. Beacham J. L. Stevenson J. C. Morris H. R. (1986). Human calcitonin gene related peptide: a potent endogenous vasodilator in man. *Clin. Sci. (Lond.)* 70 389–393 3486086 10.1042/cs0700389

[B124] Sugawara T. Ayer R. Jadhav V. Chen W. Tsubokawa T. Zhang J. H. (2011). Mechanisms of statin treatment in cerebral vasospasm. *Acta Neurochir. Suppl.* 110 9–11 21125437 10.1007/978-3-7091-0356-2_2

[B125] Sullivan G. W. Sarembock I. J. Linden J. (2000). The role of inflammation in vascular diseases. *J. Leukoc. Biol.* 67 591–602 10810997 10.1002/jlb.67.5.591

[B126] Takizawa T. Tada T. Kitazawa K. Tanaka Y. Hongo K. Kameko M. (2001). Inflammatory cytokine cascade released by leukocytes in cerebrospinal fluid after subarachnoid hemorrhage. *Neurol. Res.* 23 724–730 11680512 10.1179/016164101101199243

[B127] The ACROSS Group. (2000). Epidemiology of aneurysmal subarachnoid hemorrhage in Australia and New Zealand: incidence and case fatality from the Australasian Cooperative Research on Subarachnoid Hemorrhage Study (ACROSS). *Stroke* 31 1843–1850 10926945 10.1161/01.str.31.8.1843

[B128] Thomas J. E. Nemirovsky A. Zelman V. Giannotta S. L. (1997). Rapid reversal of endothelin-1-induced cerebral vasoconstriction by intrathecal administration of nitric oxide donors. *Neurosurgery* 40 1245–1249 9179898 10.1097/00006123-199706000-00026

[B129] Toda N. Kawakami M. Yoshida K. (1991). Constrictor action of oxyhemoglobin in monkey and dog basilar arteries *in vivo* and *in vitro*. *Am. J. Physiol.* 260 H420–H425 1996685 10.1152/ajpheart.1991.260.2.H420

[B130] Toshima M. Kassell N. F. Tanaka Y. Dougherty D. A. (1992). Effect of intracisternal and intravenous calcitonin gene-related peptide on experimental cerebral vasospasm in rabbits. *Acta Neurochir. (Wien)* 119 134–138 1481740 10.1007/BF01541797

[B131] Toyoda K. Faraci F. M. Watanabe Y. Ueda T. Andresen J. J. Chu Y. (2000). Gene transfer of calcitonin gene-related peptide prevents vasoconstriction after subarachnoid hemorrhage. *Circ. Res.* 87 818–824 11055987 10.1161/01.res.87.9.818

[B132] Tran Dinh Y. R. Debdi M. Couraud J. Y. Creminon C. Seylaz J. Sercombe R. (1994). Time course of variations in rabbit cerebrospinal fluid levels of calcitonin gene-related peptide- and substance P-like immunoreactivity in experimental subarachnoid hemorrhage. *Stroke* 25 160–164 7505492 10.1161/01.str.25.1.160

[B133] Tseng M. Y. Czosnyka M. Richards H. Pickard J. D. Kirkpatrick P. J. (2005). Effects of acute treatment with pravastatin on cerebral vasospasm, autoregulation, and delayed ischemic deficits after aneurysmal subarachnoid hemorrhage: a phase II randomized placebo-controlled trial. *Stroke* 36 1627–1632 16049199 10.1161/01.STR.0000176743.67564.5d

[B134] Tseng M. Y. Hutchinson P. J. Czosnyka M. Richards H. Pickard J. D. Kirkpatrick P. J. (2007). Effects of acute pravastatin treatment on intensity of rescue therapy, length of inpatient stay, and 6-month outcome in patients after aneurysmal subarachnoid hemorrhage. *Stroke* 38 1545–1550 17413047 10.1161/STROKEAHA.106.475905

[B135] Uddman R. Edvinsson L. Ekblad E. Hakanson R. Sundler F. (1986). Calcitonin gene-related peptide (CGRP): perivascular distribution and vasodilatory effects. *Regul. Pept.* 15 1–23 3532219 10.1016/0167-0115(86)90071-6

[B136] van Gijn J. Kerr R. S. Rinkel G. J. (2007). Subarachnoid haemorrhage. *Lancet* 369 306–318 17258671 10.1016/S0140-6736(07)60153-6

[B137] Vikman P. Beg S. Khurana T. S. Hansen-Schwartz J. Edvinsson L. (2006). Gene expression and molecular changes in cerebral arteries following subarachnoid hemorrhage in the rat. *J. Neurosurg.* 105 438–444 16961140 10.3171/jns.2006.105.3.438

[B138] Walker C. S. Conner A. C. Poyner D. R. Hay D. L. (2010). Regulation of signal transduction by calcitonin gene-related peptide receptors. *Trends Pharmacol. Sci.* 31 476–483 20633935 10.1016/j.tips.2010.06.006

[B139] Welty T. E. (1987). Use of nimodipine for prevention and treatment of cerebral arterial spasm in patients with subarachnoid hemorrhage. *Clin. Pharm.* 6 940–946 3322639

[B140] Wickman G. Lan C. Vollrath B. (2003). Functional roles of the Rho/Rho kinase pathway and protein kinase C in the regulation of cerebrovascular constriction mediated by hemoglobin: relevance to subarachnoid hemorrhage and vasospasm. *Circ. Res.* 92 809–816 12637369 10.1161/01.RES.0000066663.12256.B2

[B141] Wiley J. W. Gross R. A. Macdonald R. L. (1992). The peptide CGRP increases a high-threshold Ca^2+^ current in rat nodose neurones via a pertussis toxin-sensitive pathway. *J. Physiol.* 455 367–381 1336552 10.1113/jphysiol.1992.sp019306 PMC1175649

[B142] Wilkins R. H. (1990). Cerebral vasospasm. *Crit. Rev. Neurobiol.* 6 51–77 2225095

[B143] Winn H. R. Richardson A. E. Jane J. A. (1977). The long-term prognosis in untreated cerebral aneurysms: I. The incidence of late hemorrhage in cerebral aneurysm: a 10-year evaluation of 364 patients. *Ann. Neurol.* 1 358–370 617253 10.1002/ana.410010407

[B144] Zwienenberg-Lee M. Hartman J. Rudisill N. Muizelaar J. P. (2006). Endovascular management of cerebral vasospasm. *Neurosurgery* 59 S139–S147 17053596 10.1227/01.NEU.0000239252.07760.59

